# Challenges and Implications for Substance Use and Mental Healthcare Among Under-Resourced Women in the COVID-19 Era

**DOI:** 10.7759/cureus.62452

**Published:** 2024-06-15

**Authors:** Maria C Mejia, Alicia Kowalchuk, Sandra J Gonzalez, Muktha Nair, Lisa Webb, Nadine Scamp

**Affiliations:** 1 Population Health and Social Medicine, Florida Atlantic University Charles E. Schmidt College of Medicine, Boca Raton, USA; 2 Family and Community Medicine, Baylor College of Medicine, Houston, USA; 3 Social Work, Santa Maria Hostel, Houston, USA

**Keywords:** vaccine hesitancy, mental health, substance use, covid-19, under-resourced women

## Abstract

Background: The COVID-19 pandemic exacerbated disparities in mental healthcare and substance use disorder (SUD) treatment access, especially in under-resourced communities. This study aimed to comprehend the experiences of under-resourced women with SUD during the pandemic, their knowledge and attitudes toward it, and its impact on substance use and treatment access.

Methods: A cross-sectional study included 66 under-resourced women receiving medically managed withdrawal treatment at a community residential SUD center. Data collection occurred between November 2021 and August 2022, utilizing a 75-item instrument covering COVID-19 exposure and its impact on health, substance use, treatment access, vaccination status, beliefs, and knowledge. Descriptive analyses summarized the data.

Results: Participants faced various challenges during the pandemic. Many reported increased substance use, especially alcohol, opioids, benzodiazepines, and nicotine. Mental health stability was negatively affected, exacerbating existing disorders and limiting mental healthcare access. A majority (56.1%) reported that their chronic mental health disorder was less stable during the pandemic. Twenty (30.3%) participants reported that they had been diagnosed with a new mental health disorder since the pandemic, and 28.8% reported that it was harder for them to access mental healthcare during the pandemic. Job loss, housing instability, and financial strain were prevalent. Half (n=33, 50%) received a COVID-19 vaccine dose, while 27.3% (n=18) declined vaccination due to knowledge gaps and religious beliefs. The majority (n=41, 62.1%) worried about securing basic needs such as groceries and medication, with 64.6% (n=42) expressing a desire to cope using alcohol or drugs.

Conclusions: This study expands upon previous research by examining the effect of the COVID-19 pandemic on mental health in the context of substance use disorder treatment. Unlike previous data, which focused solely on substance use behaviors, our study delves into the impact of the pandemic on co-occurring mental health disorders. Findings underscore the need for gender-responsive and culturally appropriate SUD treatment. Vaccine hesitancy, as reflected in the study, necessitates more effective, tailored evidence-based informational campaigns. Efforts must focus on enhancing mental healthcare access, reducing stigma, and supporting individuals with co-occurring conditions amidst this evolving COVID-19 health crisis.

## Introduction

The COVID-19 pandemic has exacerbated existing disparities in access to mental healthcare and treatment for substance use disorders (SUDs), particularly in under-resourced communities [1]. The ongoing stressors and uncertainties related to the pandemic have further strained the mental and behavioral health of these communities, leading to rising rates of distress, mental illness, and substance use [2,3]. In the United States (US), the prevalence of SUDs notably increased from pre-pandemic (2019) to post-pandemic (2022). In 2019, approximately 20.4 million individuals aged 12 or older had an SUD related to alcohol or illicit drugs, constituting 7.8% of the population [2]. This included 14.5 million with an alcohol use disorder and 8.3 million with an illicit drug use disorder [2]. However, by 2022, the number rose significantly, with 48.7 million people aged 12 or older (17.3% of the population) reporting an SUD in the past year [4]. Moreover, the pandemic coincided with a surge in substance use and related fatalities. In 2021, over 106,600 deaths were attributed to drug overdose in the United States, marking the highest number on record [5]. Synthetic opioids, particularly illicitly manufactured fentanyl, have been major contributors to this increase in fatalities. The overall drug overdose death rate rose by 50% during the pandemic, with variations across states.

Additionally, the pandemic has led to several significant impacts on substance use, including increased risk of COVID-19 infection severity, increased alcohol consumption, increased infiltration of fentanyl into illicit drug supplies, and risk of drug overdose [5]. Recovery capital, which is crucial for facilitating and sustaining recovery, has also been hampered during the pandemic [6]. The personal stressors faced by those living with substance use disorders, such as job displacement, social isolation, decreased access to treatment, and disruption of support networks, have further deepened the disparities among women [7].

Women make up slightly more than half of the US population, and a significant proportion of them (15%) engage in illicit substance use [6] or have a formal diagnosis of a substance use disorder (5%) at any given time [8]. Furthermore, the COVID-19 pandemic has had a disproportionate impact on the mental health of women and low-income individuals, and this has led to an increase in alcohol consumption and alcohol use disorders among women [9,10]. Substantial increases in alcohol consumption and alcohol use disorders in women linked to disproportionate psychological distress during the pandemic are of particular concern [7-9,11]. Excessive alcohol use can lead to a variety of negative health outcomes, including liver disease, heart disease, and certain types of cancer. It can also increase the risk of accidents, injuries, and violence.

The ongoing stressors and uncertainties related to the pandemic have further strained the mental and behavioral health of these communities, leading to rising rates of distress, mental illness, and substance use. These disruptions have also increased concerns about relapse, particularly among those already in recovery [12,13]. By describing the experiences of these women, their knowledge and attitudes toward the pandemic, and the impact it has had on their substance use and access to treatment, we can better understand the unique challenges they face and work to refine substance use care delivery and recovery services to better serve this population, both now in the early post-pandemic period and in preparation for future pandemics. We aimed to describe participants' experiences with the COVID-19 pandemic, their knowledge, attitudes, and beliefs about COVID-19 and pandemic risk mitigation measures such as masking and vaccines, and the impact the pandemic has had both on their substance use and their access and engagement with treatment.

This article was previously posted to the Research Square preprint server on July 21, 2023.

## Materials and methods

This cross-sectional study was approved by the Baylor College of Medicine Institutional Review Board. We used purposive sampling from November 2021 to August 2022 to obtain the sample. We recruited 66 under-resourced women (18 years and older) with SUD receiving medically managed withdrawal treatment in a community residential SUD treatment center serving Texas Health Region 6, which includes Houston and Harris County, the most populous city and county in Texas. All instruments including the survey and interview language, as well as study processes, were designed to be trauma-informed and non-judgmental nor stigmatizing. Research team members are trained in trauma-informed care and experienced in providing clinical care to under-resourced women with substance use disorders. Informed consent was obtained from the participants, and the ethical issues involved in human research participation were adhered to in the data collection process. All the methods were carried out in accordance with relevant institutional guidelines and regulations.

The 75-item instrument covered topics regarding personal exposure to COVID-19, pandemic impact on physical and mental health status, pandemic impact on substance use and access to SUD treatment, COVID-19 vaccination status and attitudes toward COVID-19 vaccination, and knowledge, attitudes, and beliefs about the COVID-19 virus and pandemic risk mitigation measures. Demographic variables included age, gender, race, ethnicity, educational attainment, housing status, employment status, household income, parental status, and marital/relationship status. Coping strategies toward COVID-19 and COVID-19 exposure and the use of preventive measures were adapted from the CoRonavIruS Health Impact Survey (CRISIS) V0.3 and the US Centers for Disease and Control Prevention Guidance for COVID-19 [14]. The CRISIS survey is licensed on Creative Commons (CC) BY4.0 and is available for download at crisissurvey.org. The impact of COVID-19 on chronic medical conditions, substance use, and mental health questions could be answered yes, no, or don't know, and 5-point Likert scales from more, less, unchanged, unsure, and prefer not to answer. We included four questions about recognition of the clinical symptoms of COVID-19 and five questions about preventive strategies. The coping strategies and mitigation practices were answered on a 5-point Likert scale from least (1) to greatest (5).

Study data were collected and managed using Research Electronic Data Capture (REDCap) tools hosted at Baylor College of Medicine [15,16]. REDCap is a secure, web-based software platform designed to support data capture for research studies. Descriptive analyses were conducted using SPSS version 28 (IBM SPSS Statistics, Armonk, NY). Standard descriptive methods (e.g., means and standard deviations (SDs) for continuous variables, and counts and percentages for categorical variables) were used to summarize data.

## Results

Table 1 shows the characteristics of the 66 participants receiving medically managed withdrawal treatment in a community-based residential treatment center included in this study. The mean age of participants was 33.5 years (SD: 10.85 years). Most of the participants identified as cis-gendered women (n=62, 93.9%), White (n=39, 59.1%), and non-Hispanic (n=42, 63.6%). Fifty-one (77.3%) participants were unemployed, and about one-third (n=21, 31.8%) had an annual income of less than $8,000. Over half of the participants were single (n=36, 54.5%), and a majority had children (n=54, 81.8%). The average number of children was 3 (SD: 1.5), and the mean age of the children was 14.33 years (SD: 9.37 years). Education was varied but mostly between high school degree/General Educational Development (GED) and associate degree/some college.

**Table 1:** Characteristics of the study population GED: General Educational Development, SD: standard deviation

	Number	Percentage
Mean age (33.5 years) (SD: 10.85 years)	66	
Gender		
Female	62	93.9
Male	1	1.5
Non-binary	0	0
Prefer to self-describe	2	3
Prefer not to answer	1	1.5
Race		
American Indian/Alaska Native	1	1.5
Asian, Black or African American	18	27.3
White	39	59.1
Unknown	6	9.1
Prefer not to answer	5	7.6
Latinx		
Yes	20	30.3
No	42	63.6
Prefer not to answer	4	6.1
Primary language		
English	61	92.4
Spanish	3	4.5
Other	2	3
Education		
8th grade or less	2	3
9-11th grade	10	15.2
High school graduate	13	19.7
GED/high school equivalency	8	12.1
Post-high school training	8	12.1
Associate degree/some college	17	25.8
Bachelor's degree	3	4.5
Graduate or professional school	4	6.1
Unknown/prefer not to answer	1	1.5
Current employment status		
Employed	9	13.6
Unemployed	51	77.3
Supplemental Security Income	4	6
Workers' compensation	1	1.5
Prefer not to answer	2	3
Household income		
Less than $8,000	21	31.8
$8,000-$14,999	10	15.2
$15,000-$24,999	6	9.1
$25,000-$34,999	6	9.1
$35,000-$49,999	3	4.5
$50,000-$64,999	2	3
$65,000-$79,999	0	
>$80,000	2	3
Unknown/prefer not to answer	16	24.2
Marital status		
Single	36	54.5
Married/common law	15	22.7
Divorced	6	9.1
Separated	7	10.6
Widowed	2	3
Have children		
Yes	54	81.8
No	11	16.7
Prefer not to answer	1	1.5

COVID-19-/vaccine-related data

Twenty-four (36.4%) participants had had a positive COVID-19 test, and an additional 12 (18.2%) participants reported that they had personally been infected with COVID-19 but were not tested or tested negative. Of those testing positive/presumed positive, 26 (72.2%) reported that they experienced symptoms. Most participants had minor symptoms such as headache, body aches, fatigue, or runny nose/congestion. Other symptoms included fever, cough, loss of taste/smell, shortness of breath, and nausea/vomiting/diarrhea (Table 2). Seventeen (47.2%) participants sought care or treatment, mostly in an outpatient or clinic setting. Half (n=33, 50%) of the respondents received at least one dose of a COVID-19 vaccine, and most (n=49, 74.2%) did not have close personal contacts who experienced life-threatening complications (hospitalized or died) from the vaccine. There were 18 (27.3%) participants who did not receive the COVID-19 vaccine and were not considering it. In this case, respondents were asked to write their fears and concerns. Most fears and concerns centered on not having enough knowledge about the vaccine ("because it is a brand-new vaccine, and I do not trust it," "because I don't know what's in it," and "not enough research for me") and religious beliefs. Regarding sources of information, most participants (n=40, 60.6%) received information about COVID-19 from local news on TV, about half (n=34, 51.1%) from national news outlets, and a third (n=22, 33.3%) from social media. Additional sources of information were family and friends (n=18, 27.3%), healthcare providers (n=15, 22.7%), newspaper or local print media (n=10, 15.2%), and messaging platforms (n=6, 9.1%).

**Table 2:** COVID-19 exposure and symptoms COVID-19: coronavirus disease 2019

	Number	Percentage
Have you personally been infected with COVID-19?	66	
Yes, and I had a positive COVID-19 test	24	36.4
Yes, but I was not tested	12	18.2
No	30	45.5
Did you have any symptoms?	36	
Yes	26	72.2
No	9	25
Prefer not to answer	1	2.8
What symptoms did you have?	26	
Fever	17	65.4
Cough	18	69.2
Runny nose	19	73.1
Shortness of breath	14	53.8
Loss of taste/smell	18	69.2
Nausea/vomiting/diarrhea	14	53.8
Headache	21	80.8
Fatigue	19	73.1
Body aches	21	80.8
Did you seek care or get treatment?	36	
Yes	17	47.2
No	17	47.2
Prefer not to answer	2	5.6
Highest level of care setting	17	
Outpatient/clinic	9	52.9
Emergency care	5	29.4
Hospitalized but not intensive care unit	2	11.8
Intensive care unit	1	5.9
How do you think you got exposed to the virus?	36	
At work	5	13.9
Household contact	6	16.7
At school	1	2.8
While incarcerated	2	5.6
While in treatment	3	8.3
While in a social setting	12	33.3
Don't know/unsure	7	19.4
Have you been vaccinated against COVID-19?	66	
Yes, but only one dose (incomplete)	7	10.6
Yes, both doses (complete)	26	39.4
No, but considering	11	16.7
No, and not considering	18	27.3
Prefer not to answer	4	6.1

Impact on mental health

Over half of the participants (n=35, 53%) reported that they did not have any chronic medical conditions, while most (n=61, 92%) reported that they had a chronic mental health disorder.

A majority (n=37, 56.1%) reported that their chronic mental health disorder was less stable during the pandemic. Twenty (30.3%) participants reported that they were diagnosed with a new mental health disorder since the pandemic, and 28.8% (n=19) reported that it was harder for them to access mental healthcare during the pandemic.

Impact on substance use

Participants were asked how the pandemic impacted their substance use (increased, decreased, stayed about the same, slipped, or relapsed). Except for cocaine, a majority of participants indicated that substance use increased during the pandemic (compared to pre-pandemic use), including the use of opioids (n=40, 61.1%), alcohol (n=43, 65.8%), benzodiazepines (n=50, 75%), marijuana (n=36, 53.8%), tobacco/vaping/cigarettes (n=56, 84.1%), and methamphetamine (n=40, 60%). The majority (n=43, 64.6%) of participants reported that they felt a desire to cope with pandemic-influenced stressors using alcohol or drugs, 15.4% (n=10) reported no change, and 20% (n=13) reported that they did not feel a desire to cope using alcohol or drugs.

Other impacts

Many participants (n=29, 44%) reported a negative impact on their housing situation, and nearly half (n=31, 47%) reported that they lost their jobs during the pandemic. Of those who lost employment, 58.1% (n=38) received unemployment, and of the 66 respondents, 37 (56.1%) reported that they received stimulus payments, while 25 (37.9%) reported that they did not. Thirty-eight (57.6%) participants rated their financial situation as worse or less stable during the pandemic as compared to before the pandemic. A majority (n=43, 64.6%) of responders reported that their educational plans were not impacted by the pandemic.

**Figure 1:**
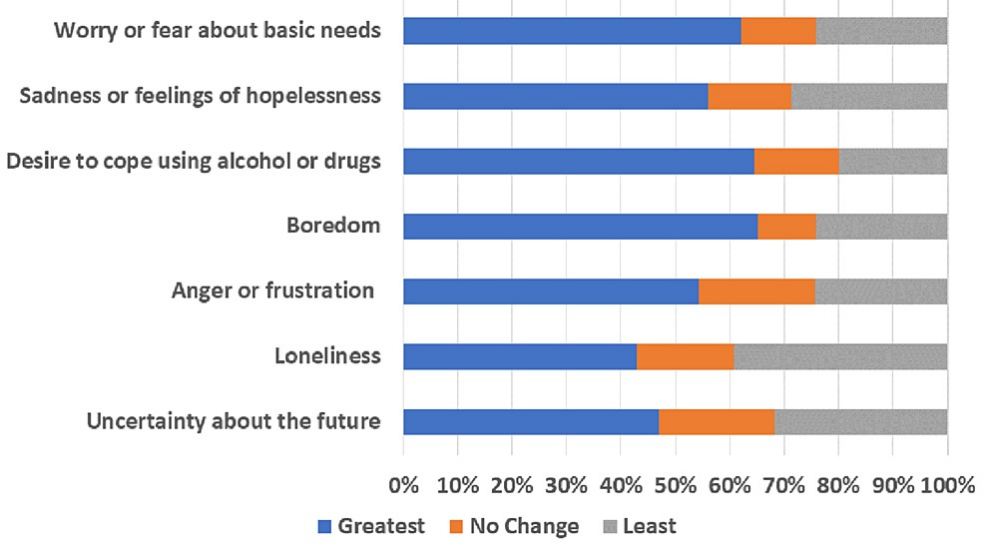
Coping strategies to deal with the stress of the COVID-19 pandemic Question: People react differently in stressful situations and may have different coping strategies to deal with the stress of an outbreak. Since the COVID-19 pandemic, have you experienced feelings of worry or fear about basic needs, sadness or feelings of hopelessness, desire to cope using alcohol or drugs, boredom, anger or frustration, loneliness, and uncertainty about the future (Likert scale from 1 (least) to 5 (greatest))? COVID-19: coronavirus disease 2019

People react differently in stressful situations and may have different coping strategies to deal with the stress of an outbreak. Figure 1 shows the participants' feelings experienced during the COVID-19 pandemic. The majority (62.1%) of the participants felt that their greatest worry was about challenges to securing basic needs such as groceries or medication, 12.6% reported no change, and 24.2% (n=16) reported that they were least worried about securing basic needs. The majority (n=41, 65.2%) of participants reported greatest feelings of boredom due to being unable to work or engage in regular daily activities and routines, 10.6% (n=7) reported no change, and 24.2% (n=16) experienced the least feelings of boredom. Over half (n=37, 56%) reported greatest feelings of sadness or feelings of hopelessness, 15.2% (n=10) reported no change, and 28.8% (n=19) reported sadness or feelings of hopelessness were least. Similarly, about half (n=36, 54.4%) reported greatest feelings of anger or frustration, 21.2% (n=14) reported no change, and 21.2% (n=14) reported that their feelings of anger or frustration were least concerning. Less than half of the participants reported greatest feelings of loneliness (n=24, 36.4%) and uncertainty about the future (n=31, 47%).

**Figure 2:**
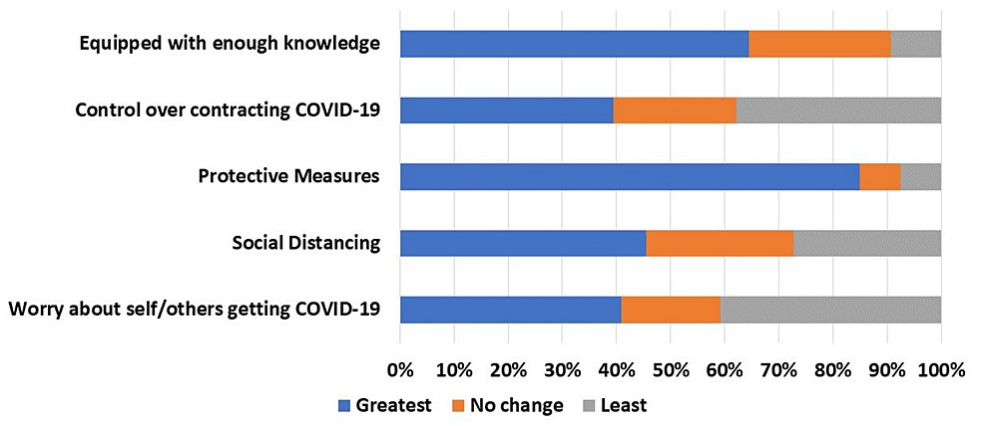
COVID-19 exposure and use of preventive measures Questions: Do you feel like you are equipped with enough knowledge to protect yourself and your household from COVID-19? How much control do you feel like you have over whether or not you (or a member of your household) might contract COVID-19? How often do you practice protective measures such as hand washing, and using hand sanitizer? How much social distancing do you practice? How worried are you about you or individuals in your household getting COVID-19 (Likert scale from 1 (least) to 5 (greatest))? COVID-19: coronavirus disease 2019

The majority (n=43, 64.6%) of the participants felt that they were equipped with enough knowledge to protect themselves and their household from COVID-19 (Figure 2). Most (n=56, 84.8%) were using protective measures such as hand washing and using hand sanitizer, while fewer (n=30, 45.4%) were practicing social distancing. Responses to the question, "How worried are you about you or individuals in your household getting COVID-19" elicited mixed results with an equal number (n=27, 40.9%) reporting that they were most worried or least worried, and 18.2% (n=16) reported no change. Similar responses were reported about the degree to which they felt able to control whether they (or a member of their household) might contract COVID-19.

## Discussion

Substance use disorder treatment should consider not only biological differences between genders but also social and environmental factors that may influence motivations for drug use, reasons for seeking treatment, types of environments where treatment is obtained, treatments that are most effective, and consequences of not receiving treatment [17]. For instance, social and cultural norms, stigma, and discrimination may influence whether and how women seek treatment for substance use disorders. Women may also face additional barriers to accessing treatment, such as childcare responsibilities, transportation issues, and financial constraints. Therefore, gender-responsive and culturally appropriate approaches to substance use disorder treatment are crucial to address the unique needs and challenges faced by women with substance use disorders. Such approaches may involve integrating gender-specific interventions, providing trauma-informed care, addressing co-occurring mental health conditions, and involving family members and social support networks in the treatment process.

Women with substance use disorders have a higher prevalence of mental and behavioral health disorders compared to the general population [18]. This study expands upon previous research [12] by examining the effect of the COVID-19 pandemic on mental health in the context of substance use disorder treatment. Unlike previous data, which focused solely on substance use behaviors, our study delves into the impact of the pandemic on co-occurring mental health disorders. In this study, most participants (56.1%) reported a chronic mental health disorder that was less stable during the pandemic, and 28.8% reported that it was harder for them to access mental healthcare during the pandemic. Our findings highlight the challenges faced by individuals with chronic mental health disorders during the COVID-19 pandemic. The pandemic disrupted the delivery of mental health services and caused significant stress and anxiety for many individuals, including those with pre-existing mental health conditions. The difficulty in accessing mental healthcare during the pandemic can be attributed to several factors, including closures of mental health facilities, limitations on in-person services, and disruptions in insurance coverage [5,6]. It is important to recognize the impact of the pandemic on mental health and provide resources and support to help individuals manage their mental health conditions during this early post-pandemic period and in future global crises leading to similar disruptions.

Co-occurring mental health conditions are common among individuals with substance use disorders and can complicate treatment and recovery. Women with SUD are more likely to experience depression, anxiety, post-traumatic stress disorder, and other trauma-related disorders compared to men with SUD. Additionally, women with SUD may have a higher risk of experiencing interpersonal violence, which can contribute to the development of mental health conditions. A recent study reports a more than twofold increase in intimate partner violence homicides in the geographical location where the study was conducted [19].

During the COVID-19 pandemic, a public health crisis with global societal impacts, nearly two-thirds of this study's participants reported a significantly increased desire to cope with stressors through substance use, and the use of all substances increased, except for cocaine. Data shows that people with SUD, especially individuals with opioid use disorder and African Americans, may be at higher risk of COVID-19 infection and poorer outcomes [20]. Higher psychological distress in men and women related to coping with COVID-19 and SUD [12] and increased alcohol use as a coping skill [8] have also been linked to the pandemic. People with lower incomes are generally more likely to report major negative mental health impacts from worry or stress over COVID-19.

While in this study, nearly half of all participants who had been diagnosed or with presumed COVID-19 infections reported having mostly minor, self-limiting symptoms, having a substance use disorder can increase the likelihood of severe illness from COVID-19 [20]. People who use drugs may also have underlying medical conditions that put them at increased risk for severe illness from COVID-19 [21]. Increased risk of SARS-CoV-2 breakthrough infections has been linked to co-occurring health conditions and adverse socioeconomic determinants of health, which are more common in people with substance use disorders [22]. People with substance use disorders, such as alcohol, cannabis, cocaine, opioid, and tobacco use disorders, also had elevated rates of severe outcomes, including hospitalization and death, following breakthrough infections.

It is essential to provide information that is directed and tailored to under-resourced populations to address the inequities, meet the public mental health and substance use disorder treatment needs, and improve services. Study participants reported receiving most of their information about COVID-19 from local and national televised news services, and nearly two-thirds felt that they had received sufficient information to protect themselves and their households from COVID-19. Over 80% reported using some mitigation strategies, half had received at least one dose of a COVID-19 vaccine, and just over a quarter of participants had not been vaccinated and were not planning to be, most commonly due to lack of enough information on the vaccines or for religious reasons. Individuals with substance use disorders may face unique challenges that prevent them from adhering to public health recommendations, such as limited personal resources, unstable and densely populated housing conditions, substance use sharing practices, and compromised immunity [23]. Therefore, it is crucial to develop and disseminate information that is accessible and culturally relevant and addresses the specific challenges faced by these populations. This may involve partnering with community-based organizations, using culturally appropriate messaging and media channels, and providing resources and support to help individuals with substance use disorders access care and adhere to public health guidelines. Additionally, addressing social determinants of health, such as housing, employment, and access to healthcare, is critical to improving the health outcomes of under-resourced populations with substance use disorders.

Individuals with SUDs, including those in long-term recovery, and their families continue to face challenges to SUD treatment and recovery support services due to changes and uncertainty related to treatment service disruptions such as face-to-face versus telehealth appointments, access to medications, and changes in prescribing practices [24-26]. Already marginalized populations, such as pregnant and postpartum women with SUD, struggled to gain access to SUD services prior to the pandemic [27-29]. Pandemic-related restrictions faced by treatment facilities include a temporary shutdown of services to pivot to teleservices, suspension or reduction of residential admissions, overwhelmed staff, and participants leaving treatment for fear of contracting COVID-19 [26,29,30].

The study has some limitations to consider, including the potential for self-reporting and recall bias as it relies on individuals' willingness and ability to provide accurate and honest information about their substance use and related behaviors. However, our research team has extensive training in trauma-informed care and experience establishing rapport and trust with participants. In addition, to mitigate these limitations, we established safeguards ensuring confidentiality and anonymity. Furthermore, surveys conducted among women with SUDs often face challenges in achieving a representative sample. Factors such as limited access to treatment, differential engagement in healthcare or research, or the exclusion of certain subpopulations (e.g., those involved in the criminal justice system) can introduce sampling bias and limit the generalizability of findings.

The COVID-19 pandemic has starkly highlighted the systemic inequities faced by individuals with SUDs, especially women from under-resourced communities. Proactive measures are essential to address these disparities and ensure equitable access to care in future health crises.

One key aspect to consider is the intersectional nature of health disparities, which are shaped by a complex interplay of social, economic, and environmental factors. By adopting a comprehensive, intersectional approach to health equity, stakeholders can better address the underlying determinants of health disparities and ensure equitable access to care for all individuals, regardless of socioeconomic status or demographic characteristics. This approach requires collaboration across sectors, including healthcare, social services, and community organizations, to address the multifaceted needs of under-resourced populations and build more resilient communities, ensuring that no one is left behind in times of crisis.

Moreover, innovative solutions are needed to enhance the resilience of healthcare systems and improve the delivery of services to underserved populations. Telehealth services, community-based interventions, and peer support programs have emerged as vital tools for expanding access to care, particularly in remote or resource-limited settings. By leveraging technology and community resources, healthcare providers can bridge gaps in care and reach individuals who may face barriers to traditional healthcare services. Additionally, addressing the underlying social determinants of health, such as housing instability, food insecurity, and unemployment, is essential for advancing health equity and reducing disparities in SUD treatment access and outcomes.

## Conclusions

As the world grapples with a resurgence of COVID-19 cases, heightening its potential to escalate into an extended epidemic, the challenges faced by under-resourced women with SUD continue to prevail. The pandemic heightened their stress levels, exacerbated mental health issues, and led to an uptick in substance use. Notably, while many were aware of how to protect themselves from the virus, significant barriers to accessing essential SUD and mental health treatments prevailed. Alarmingly, half remained unvaccinated, driven by a combination of insufficient information about vaccines and religious beliefs. This current spike in cases and the looming epidemic potentially underscore the pressing need for a rapid, concerted response. Primary among the priorities is addressing the glaring disparities in SUD treatment, emphasizing the necessity to make treatments not only accessible but also affordable for these marginalized communities. The design of these treatments should be sensitive to the unique gender and cultural challenges faced by these women. With mental health taking a significant hit during the pandemic, the integration of mental health solutions into SUD treatments becomes even more critical.

Given the added adversities that the pandemic has layered onto these women, from housing crises to job losses, strengthening their social support networks is indispensable. Vaccine hesitancy, as reflected in the study, necessitates more effective, evidence-based informational campaigns, complemented by efforts to reduce stigmas around both SUD and mental health. In this challenging landscape, expanding the reach and accessibility of health services becomes pivotal. Implementing these measures will not only shield these vulnerable groups from the devastating impacts of the current surge but will also serve as a solid foundation for ensuring they receive the holistic, comprehensive care they so urgently need amidst this evolving health crisis.
